# Self-Attitude and Reflection in Codependent Women: A Comparative Study

**DOI:** 10.11621/pir.2024.0107

**Published:** 2024-03-15

**Authors:** Anastasya S. Kolenova, Anna M. Kukulyar, Ekaterina G. Denisova, Pavel N. Ermakov

**Affiliations:** a *Southern Federal University, Rostov-on-Don, Russia*; b Don State Technical University, Rostov-on-Don, Russia

**Keywords:** codependency, codependent women, self-attitude, reflection, codependent behavior, addictive behavior, alcoholism, drug addiction, non-chemical addiction

## Abstract

**Background:**

Currently, psychological knowledge integrates theories on codependent behavior, advancing our understanding of this phenomenon. However, empirical research is lacking to understand its specific manifestations in relation to different types of addiction.

**Objective:**

To identify the features of self-attitude and reflection in codependent women.

**Design:**

The study included 233 women (ages 18–70), including 102 in relationships with a person addicted to alcohol, drugs, or suffering from a non-chemical addiction). The study was conducted using the following methods: Self-Attitude Questionnaire (Stolin & Panteleev, 1988); Differential Type of Reflection Questionnaire (Leontiev, 2009); and Codependency Self-Inventory Scale (CSIS; Weinhold & Weinhold, 1989; translated by A. G. Cheslavskaya, 2002).

**Results:**

Significant differences were observed in self-attitude and reflection. Women with high codependency have lower self-esteem and tend to underestimate their abilities, compared to the control group. They also exhibit a lower sense of control over their lives, lower self-consistency; they tend to focus on their weaknesses and shortcomings and are more inclined to self-blame. Quasi-reflexivity is more common in codependent women who have relationships with non-chemical addicts or do not identify an addict in their lives.

**Conclusion:**

There are significant differences in self-attitude and reflection between the control group and subgroups of women in relationships with different types of addicts; the same differences were observed in comparison of women with low and high codependency levels. The study contributes to a better understanding of the psychological mechanisms underlying codependent behavior in women and has implications for future research and clinical practice.

## Introduction

Codependency is a phenomenon that manifests itself in dependent behavior, due to a change in value-semantic constructs, a lack of necessary competencies, and is formed under the influence of the negative experience of dysfunctional relationships with significant others (Kolenova et al., 2023).

### Self-Attitude Studies in Codependent Individuals

Previous studies have generally supported the idea that self-attitude and reflection can play a significant role in shaping the self-concept of a codependent personality (Emelyanova, 2008; Khazova & Shipova, 2020; Khazova & Varioshkina, 2022; Vasenkin, 2020). This suggests that the way individuals with codependency perceive themselves is influenced by the specific attitude they have toward themselves and how they engage in thinking about and understanding themselves. In the psychological literature, in the most general sense, self-attitude is commonly understood as the specificity of an individual’s attitude towards their “Self” (Meshcheryakova & Zinchenko, 2007). Also, self-attitude is considered an integral self-assessment of particular aspects, weighted by their subjective significance (Sargeveladze, 2000). Self-attitude is also understood as a feeling towards “the Self”, including experiences of various kinds (self-confidence, self-acceptance, self-sympathy, reflected attitude, etc.) (Petrovsky & Yaroshevsky, 1990).

The study of self-attitude as a single research object most often highlights two aspects in a single process of self-consciousness: the process of gaining knowledge about oneself (self-awareness and awareness of this knowledge) and the process of forming an attitude towards oneself (together with a relatively stable attitude towards oneself as a certain long-term characteristic) (Ermolenko, 2016).

In our study, we adopt the framework of V.V. Stolin and S.R. Panteleev, which posits that “individual self-assessments of personal qualities are not directly linked to overall self-attitude. Instead, they are organized at an intermediate level determined by the spheres of the subject’s life or their personal manifestations. Self-attitude, as a hierarchical structure, encompasses private self-evaluations, the integration of personal phenomena into spheres, and a complex formed by the generalized ‘Self’ positioned at the top of the hierarchy. The integral indicator represents a global scale of self-attitude, relatively autonomous and one-dimensional, reflecting a certain generalized characteristic equally relevant to various spheres of the Self” (Stolin & Panteleev, 1988).

Empirical and theoretical studies analyzing the self-concept of codependent individuals have shown a significant reduction in self-esteem and self-attitude (Kogan et al., 2012; Moskalenko, 2008; Stryapukhina & Posokhova, 2022; Tulebaeva, 2017). There is also evidence of a lack of psychological boundaries and a tendency to violate those of others, negative attitudes towards oneself and others, and a lack of personal resources (Khazova & Varioshkina, 2022). Additionally, codependent individuals tend to ignore their own needs (Kolenova, 2019; Moskalenko, 1994), experience auto- and heterogeneous anger, and exhibit negative generalization of self-relationship, among other things (Tascheva & Bedredinova, 2016). Rogozina (2020) has shown that the degree of marital codependency is closely related to the structure of the self-relationship, and it can be assumed that the considered components of the self-relationship play a significant role in the formation and degree of marital codependency in women. Lampis et al. (2017) demonstrated that codependent individuals experience a disruption in the differentiation of the self (i.e., self-positioning, emotional reactivity, emotional detachment, merging with others), as well as a transformation of the self-relationship. Bacon et al. (2020) noted the absence of clear self-awareness, a stable pattern of extreme emotional imbalance, imbalanced relationships, and occupational imbalance among codependent individuals. Due to their vulnerability, shame, low self-esteem and self-acceptance, codependent individuals develop an attachment style that is more or less anxious or avoidant. They develop anxious and avoidant attachment styles, and exhibit behaviors that are either pursuing or distancing (Erol & Ort, 2014).

### Reflection Studies in Codependent Individuals

Various approaches to understanding reflection can be distinguished today, including activity-based, personality-based, pedagogical, metacognitive, and other directions of research (Karpov, 2004; Kayasheva, 2014; Kayasheva & Khanova, 2019; Medvedev et al., 2020; Rostovtseva & Guseva, 2021). Such a diversity of approaches in Slavic literature, on the one hand, refl ects the wide use of the term and promotes a multifaceted understanding of the problem of reflection. On the other hand, it complicates the comprehension of this psychological category. In our study, we use D.A. Leontiev’s model of personality reflexivity, distinguishing four foci of the direction of consciousness: on an external intentional object (lack of reflective processes), on the subjects themselves (introspection), on themselves and the object simultaneously (systemic reflexivity), and on external objects beyond the actual situation (quasi-reflexivity) (Leontiev & Osin, 2014). This model helps us to understand various aspects of reflexivity and how individuals engage in reflective processes.

There is a limited number of studies examining the reflection of codependents. Kayasheva and Efremova (2015) found that personal reflection enhances the stress resistance of codependent women. It was also found that women who do not suffer from codependency exhibit a higher level of constructive personal reflection compared to codependents. In another study, Kayasheva and Efremova (2016) discovered that more pronounced violations of temporal, emotional, and cognitive characteristics in codependent women result in lower values for life orientation and personal reflection. They reported higher manifestations of protective mechanisms such as repression, regression, substitution, projection, compensation, hypercompensation, coping strategies of confrontation and escape/avoidance, as well as higher levels of subjective feelings of loneliness and depression. While they investigated the subjective experience of divorce, there is currently no research on the specifics of self-attitude and reflection in codependent women, taking into account the specifics of the dependent behavior of a partner or relative who is addicted to alcohol, drugs, or suffering from non-chemical addiction. Therefore, the present study aims to explore the characteristics of reflection in codependent women concerning the level of codependent behavior in a routine situation, which will provide insight into the aspects of reflection that predominate in codependent women. Studying the specifics of self-attitude and reflection in codependent women is both relevant and novel.

The present study aims to investigate the specificities of self-attitude and reflection in codependent women. The research hypothesis is that the self-attitude and reflection of codependent women may have unique features, notably that the characteristics of self-attitude and reflection may differ not only between the experimental and control groups, but among groups of codependent women based on the type of addiction of their partner or relative.

## Methods

### Participants

The study involved 233 women aged 18 to 70 years, including 102 who currently are in a romantic relationship or are related to an addict (suffering from alcoholism, drug addiction, or non-chemical addiction) and 131 women who have not identified a partner or relative with an addiction.

### Procedure

Psychological testing was conducted in person, at several psychological centers that provide psychological assistance to addicts and their families. All participants were offered standardized electronic forms for testing and questionnaires. All respondents participated voluntarily. Participants were asked to provide information on gender, age, type of addiction, and their relationship with the addict.

Those participants who reported having a relative or a partner with an addiction constituted the main study group. Respondents who did not report having an addict in their lives (as a partner or as a relative) comprised the control group. The experimental and control groups were also divided into 5 subgroups:

1. Codependents-alcoholism — subgroup 1 — 52 women who are in a relationship with or are related to an alcoholic.
2. Codependents-drug addiction — subgroup 2 — 22 women who are in a relationship with or are related to a drug addict.
3. Codependents-non–chemical addictions — subgroup 3 — 28 women who are in a relationship with or are related to a non-chemical addict (various forms of gambling addictions and other behavioral addictions).
4. Subgroup 4, with high codependency on the Codependency Self-Inventory Scale — 68 women who did not identify a partner or relative suffering from an addiction, but who have high codependency according to the testing, which allows us to assume that the pattern of codependency is manifested in this group of respondents in the interpersonal sphere.
5. Control group, subgroup 5 — 63 women who did not identify an addicted partner or relative in their anamnesis and have a low or medium level of codependency according to the results of psychological testing.

#### Questionnaires

The following psychological tests were employed in the study:

*The Self-Attitude Questionnaire* by V.V. Stolin and S.R. Panteleev (1988), which was developed in 1985, comprising 57 statements requiring unequivocal agreement or disagreement; the statements are grouped into seven scales and four integral indicators (Cronbach’s α is .83).

*The Differential Type of Reflection Questionnaire* by D.A. Leontiev and E.N. Osin (2014), which was developed in 2009, consisting of 30 statements for which the subject was to express the degree of agreement or disagreement; the statements are grouped into three scales (Cronbach’s α is .79).

*The Codependency Self-Inventory Scale (CSIS)* by B. Weinhold and J. Weinhold, developed in 1989, adapted and translated into Russian (Kocharyan et al., 2006), containing 20 items describing behavior patterns, with respondents evaluating the frequency of such behavior; statements are grouped into seven scales and four integral indicators (the criterion validity of the method is 0.38).

#### Statistical Analysis

We used the Shapiro-Wilk test to determine whether the empirical distribution is normal. To investigate the significance of differences in the selected subgroups, we applied the nonparametric Kruskal-Wallis test with Dunn’s post-hoc pairwise comparison. The JASP 0.16 soft ware package was used for statistical data analysis.

## Results

A preliminary examination of the data distribution for the scales revealed deviations from normality, leading us to use the nonparametric Kruskal-Wallis test to investigate differences among the subgroups. With respect to our hypothesis regarding specific aspects of self-attitude and reflection in the codependent women, we performed a comparative analysis using the Kruskal-Wallis test. *Table 1* presents the results of this analysis for the selected groups and the control subgroup (subgroup 5).

**Table 1:** Results of comparative analysis (Kruskal-Wallis test)

	Average values in the studied subgroups					Kruskal-Wallis Test	*p-*value
	Subgroup 1	Subgroup 2	Subgroup 3	Subgroup 4	Subgroup 5		
Scale (global S self-attitude)	18.3	17.4	18.3	18.9	21.8	38.5	< .001
Self-respect (I)	9.3	8.3	9.1	9.4	11.4	30.2	< .001
Auto-sympathy (II)	9.1	8.2	9.2	9.2	11.2	35.1	< .001
Expected from others attitude (III)	9.1	8.9	8.9	8.8	9.4	5.2	.27
Self-interest (IV)	6.7	6.9	6.8	7.0	7.3	7.7	.104
Self-confidence	5.0	4.7	4.8	4.9	5.8	19.5	< .001
Attitude of others	5.6	5.7	5.7	5.6	5.8	3.1	.542
Self-acceptance	4.7	4.6	4.5	4.8	5.2	9.7	.046
Self-management	4.7	4.1	4.4	4.6	5.2	17.8	< .001
Self-blame	4.1	4.7	4.0	4.0	2.8	28.5	< .001
Self-interest	6.1	5.9	6.1	6.1	6.5	15.5	.004
Self-understanding	4.1	3.7	4.4	4.3	5.0	24.1	< .001
System reflection	40.6	39.9	41.8	40.2	38.7	8.9	.063
Introspection	23.3	24.3	24.0	24.9	19.2	32.4	< .001
Quasi-reflection	24.6	25.4	26.4	26.9	22.4	24.4	< .001

Significant differences were found in almost all the scales between the control group (with low codependency) and respondents with identified codependent behavior, including the “S Scale (global self-attitude)”, “Self-esteem”, “Auto-sympathy”, “Self-confidence”, “Self-leadership”, “Self-blame”, “Self-understanding”, “Introspection”, and “Quasi-refl ection” (*p* < .001). Differences with lower levels of statistical significance were also found in “Self-acceptance” and “Self-interest” (*p* < .05).

To further analyze the identified differences, we conducted an a posteriori analysis of pairwise comparisons using the Dunn’s test and Holm’s correction.

This comparison allowed us to specify the differences in self-attitude and reflection characteristics not only between the experimental and control groups, but also between groups of codependents depending on the type of addiction of their partner or relative.

**Figure 1.**
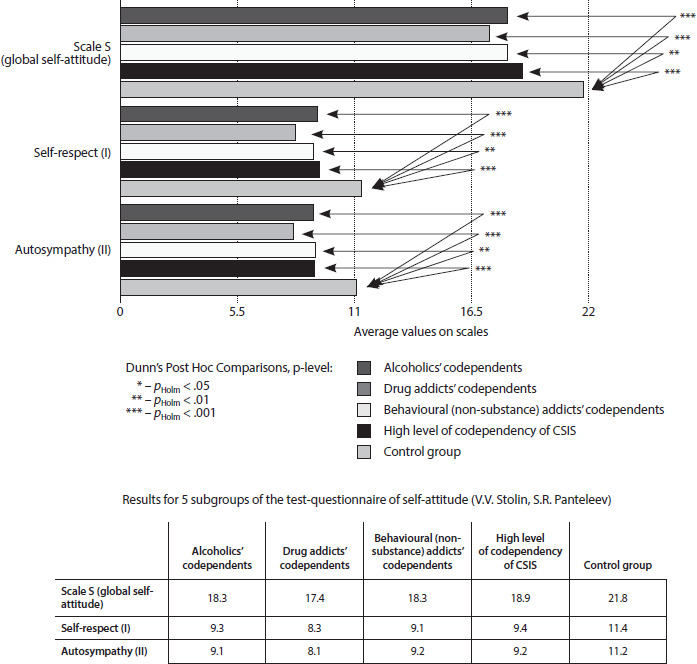
Differences among the five subgroups: post hoc pairwise comparisons for integrative self-attitude indicators

Pairwise comparison using the Dunn’s test showed significant differences on integrative self-attitude indicators of the Self-Attitude Questionnaire (Stolin and Panteleev, 1988) between the control group and all other groups (see *Figure 1*). The S scale (global self-attitude) varies significantly between the control group and the high codependency on the CSIS subgroup (*z* = –4.7, *p*_Holm_ < .001), Codependents-alcoholism (*z* = 4.9, *p*Holm < .001), Codependents-drug addiction (*z* = 4.5, *p*_Holm_ < .001), Codependents-non-chemical addictions (*z* = 3.6, *p*_Holm_ = .001). The control group has higher global self-attitude, while the Codependent groups (subgroups 1, 2 and 3) have the lowest scores.

The Self-respect scale showed significant differences between the control group and the group with high codependency (*z* = –4.3 with *p*_Holm_ < .001), the group of Codependents-alcoholism (*z* = 4.0 with *p*_Holm_ < .001), the group of Codependents-drug addiction (*z* = 4.2 with *p*_Holm_ < .001), the Codependents of non-chemical addicts (*z* = 3.2 with *p*_Holm_ = .005). The control group had higher Self-respect (11.4) and the subgroup with high codependency on the CSIS had the lowest Self- respect (8.3) among the studied groups.

The auto-sympathy scale showed significant differences between the control group and the group with high codependency (*z* = –4.6 with *p*_Holm_ < .001), the group of Codependents-alcoholism (*z* = 4.3 with *p*_Holm_ < .001), the group of Codependents-drug addiction (*z* = 4.7 with *p*_Holm_ < .001), the group of Codependents-non-chemical addictions (*z* = 3.1 at *p*_Holm_ = .008). The control group had significantly higher auto-sympathy (11.2), while the group of Codependents-drug addiction had the lowest level (8.2) among the groups studied.

**Figure 2.**
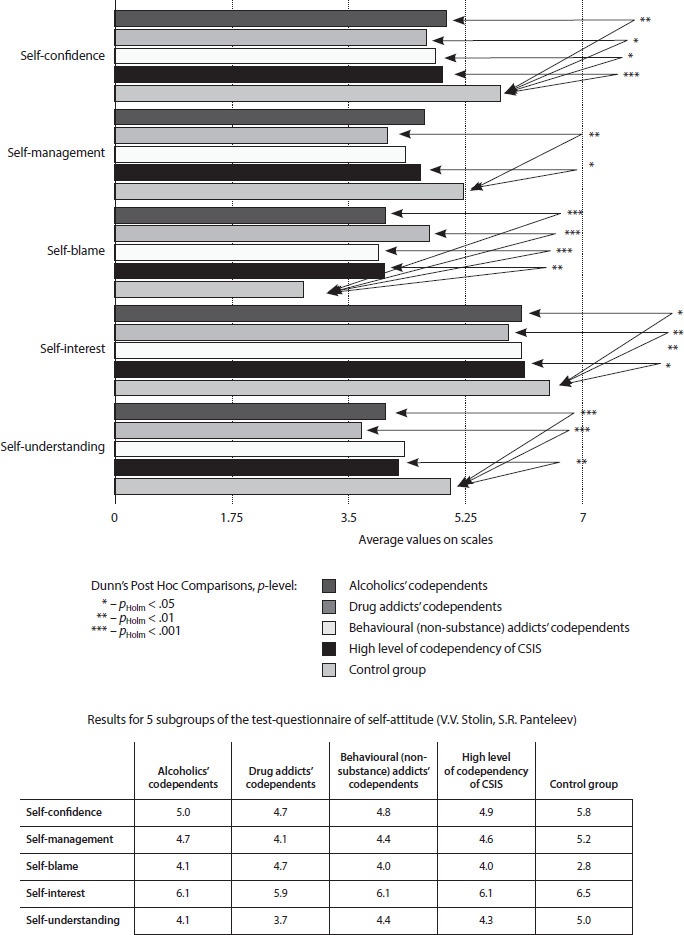
Differences among the five subgroups: post hoc pairwise comparisons for internal actions of self-attitude (specific attitudes towards one’s self)

Pairwise comparison showed significant differences on almost all indicators of the Self-Attitude Questionnaire (Stolin and Panteleev) between the control group and all other subgroups (see *Figure 2*). Significant differences on the Self-confidence scale were found between the control group and subgroup 4 with high codependency (*z* = –3.8 with *p*_Holm_ < .001), subgroup 1 of Codependents-alcoholism (*z* = 3.2 with *p*_Holm_ = .006), subgroup 2 of Codependents-drug addiction (*z* = 3 with *p*_Holm_ = .010), and subgroup 3 of Codependent-non-chemical dependencies (*z* = 2.663 at *p*_Holm_ = .027). The Self-management scale also showed significant differences between subgroup 4 and the control group (*z* = –3.1 at *p*_Holm_ = .010), and the subgroup of Codependents-drug addiction and the control group (*z* = 3.7 at *p*_Holm_ = .001).

The Self-blame scale showed significant differences between the control group and the high codependency subgroup (*z* = 4.1, *p*_Holm_ < .001), as well as the Codependents-alcoholism subgroup (*z* = –3.8, *p*_Holm_ < .001), the Codependents-drug addiction subgroup (*z* = –4.3, *p*_Holm_ < .001), and the Codependents-non-chemical dependencies subgroup (*z* = –2.8, *p*_Holm_ = .019).

On the Self-interest scale, there were significant differences between the control group and the subgroup with high codependency (*z* = –2.6 at *p*_Holm_ = .037), subgroup 1 of Codependents-alcoholism (*z* = 3 at *p*_Holm_ = .012), and subgroup 2 of Codependents-drug addiction (*z* = 3.2 at *p*_Holm_ = .006) (*see Figure 2*). Likewise, significant differences on the Self-understanding scale were found between the subgroup with high codependency and the control group (*z* = –3.4 at *p*_Holm_ = .003), in subgroup 1 of Codependents-alcoholism and the control group (*z* = 3.7 at *p*_Holm_ < .001), and in subgroup 2 of Codependents-drug addiction and the control group (*z* = 4.1 at *p*_Holm_ < .001).

**Figure 3.**
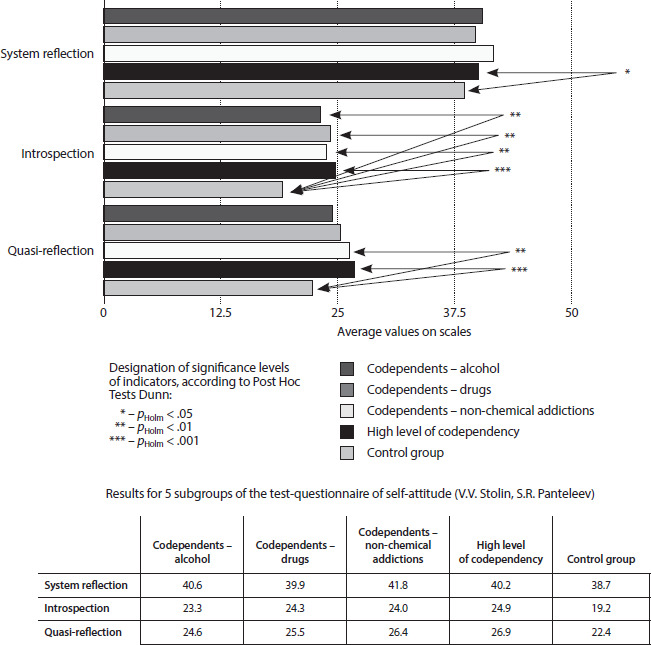
Differences among five subgroups: post hoc pairwise comparisons for types of reflection

Pairwise comparison showed significant differences on the scales of the “Differential Type of Refl ection” questionnaire (Leontiev) (see *Figure 3*). Subgroup 3 of Codependents-non-chemical addictions showed the highest level of “Systemic reflection” (41.8), while the control group had the lowest level (38.7). However, significant differences with correction for multiple comparisons were observed between the control group and the group with high CSIS scores (*z* = –2.6 at *p*_Holm_ = .044).

The “Quasi-reflection” scale also showed significant differences between the control group and subgroup 4 with high codependency (*z* = 4.7 with *p*_Holm_ < .001), as well as with subgroup 3 of Codependents-non-chemical dependencies (*z* = –3.3 with *p*_Holm_ = .004). Here the control group showed the lowest quasi-reflection (22.4) and the high codependency group had the highest levels (26.9) among the subgroups studied.

On the “Introspection” scale, the control group showed significant differences with subgroup 1 of Codependents-alcoholism (*z* = –3.6 at *p*_Holm_ = .001), subgroup 2 Codependents-drug addiction (*z* = –3.5 at *p*_Holm_ = .002), subgroup 3 Codependents-non-chemical dependencies (*z* = –3.7 with *p*_Holm_ = .001), and subgroup 4 with high codependency (*z* = 5.2 with *p*_Holm_ < .001). The last one showed the highest score (24.9) and the control group had the lowest (19.2).

The results partially confirm the hypothesis, as significant differences were found among the groups. The mean values differed between codependent groups with different types of addicts and the control group. However, the differences mainly concern the specific characteristics of self-attitude and reflection of codependent women (subgroups 1-4) compared to women who have low codependency and are not in a relationship with addicts (control group). Additionally, the quantity and nature of differences between the subgroup with high codependency and the control group are similar to those between the control group and subgroups of codependents who have identified addicts in their lives.

## Discussion

The study found that codependent women exhibit distinct characteristics of self-attitude and reflection compared to women with low codependency and no addicts in their lives (control group). Furthermore, these parameters varied depending on the addiction type of their partner or relative, which may determine the specifics of the relationship with the dependent, but no significant differences were found among these groups, possibly due to the relatively small sample size, especially in subgroups 2 and 3. Significant differences were observed in the self-image and self-attitude of codependent women, including greater categoricity in self-assessment, less faith in their abilities, underestimation of capabilities, low self-esteem, and a self-blame tendency, which is consistent with previous studies (Andronnikova & Perminova, 2017; Dmitrieva & Raklova, 2004; Karpushina, 2017; Lampis et al., 2017; Merinov et al., 2015; Polkova, 2018; Rogozina, 2020). In Rogozina’s study (2020), it was confirmed that the level of codependency is linked to the self-attitude of codependent women, namely the level of marital codependency has a positive association with self-blame and inverse associations with such indicators as global self-attitude, self-understanding, self-confidence, expected attitude of others, and self-acceptance. These findings suggest that the predominant development of positive traits in self-attitude indicates a self-suffcient and self-confi dent personality with lower codependency. Similar results on self-concept in codependent women are also reported by foreign colleagues, including Lampis et al. (2017), Bacon et al. (2020), Orbon et al. (2021), and Rodríguez et al. (2013).

Upon closer examination of the specific attitudes towards internal actions, distinctive features were found in codependent women, depending on their partner’s/ relative’s addiction type. Women with close relationships with alcohol addicts exhibited low self-confidence, low appreciation of self-interest and understanding, and a greater tendency to self-blame, which aligns with previous research (Abakumova et al., 2017; Chernobrovkina, 2009; Politika, 2020). These trends are similar to those of women with relationships with drug addicts, except for the diffculty in explaining their actions to themselves, leading to a lesser sense of understanding. Women who have close relationships with individuals with non-chemical addictions exhibit a tendency for self-blame and self-flagellation, as well as low confidence in their interest in others (Zrazhevskaya et al., 2020).

It is noteworthy that a group of women who exhibited high codependent behavior despite having no history of an addicted partner or relative showed tendencies seen in all the previously mentioned codependent groups. These tendencies include lower self-confidence, self-understanding, self-management, self-interest, and higher self-blame. This observation suggests that codependent behavior in women may lead to changes in personal components, particularly in the dynamics of the self-image.

All groups of codependent women show active self-reflection, indicating a focus on their own experiences, which can lead to a tendency to dissociate, obsessing over their thoughts. However, codependent women who have relationships with non-chemical addicts or do not specify their relationship with an addict exhibit greater quasi-reflection, suggesting a focus on an unrelated object and separation from reality. This finding supports the idea of hypercompensation, which is consistent with the findings of A.A. Berdichevsky (2019). The subgroup of codependents in relationships with non-chemical addicts showed the highest level of systemic reflection. This form of reflection is the most adaptive, as it provides a comprehensive view of the interaction situation, including both the subjective and objective poles. Therefore, it is possible to suggest that in a relationship with a behavioral addict, codependent women may have a clearer view of their own behavior and a tendency towards self-determination and effective adaptation.

Overall, our findings on the specific expression of self-attitude traits in codependent women are consistent with other correlational studies (Khazova & Varioshkina, 2022; Khlebnikova, 2020; Kuhtova & Antonova, 2022; Mokinoy, 2018; Puchkova & Stadnik, 2020; Varioshkina, 2015, etc.). The scientific novelty and value of the results is that some features of self-attitude and reflection in women were identified depending on the severity of their codependence, and the main differences were determined depending on the type of addiction of their partner or relative. Previous research has tended to focus on the general characteristics of codependency with addicts, without considering the type of addiction. Therefore, although pairwise comparison did not confirm this part of the hypothesis, the results of this study provide valuable information and may lead to a more differentiated approach to the treatment and management of women with codependency.

## Conclusion

In this study, we aimed to discover the unique characteristics of self-attitude and reflection in codependent women. Our results showed significant differences in both self-attitude and reflection between the control group and women with high codependency, as well as women who have relationships with or are related to different types of addicts. Our data suggest that reflection, self-blame, and self-management of codependents are likely to be influenced by the type of addiction. However, further research with a larger sample and expanded research subject is needed to more fully understand the impact of addiction on the personality and behavior of codependent women.

Future research could also narrow or broaden the age range of the codependent women, to study other possible psychological and social factors. It could focus on more specific age ranges and explore how self-attitude and reflection vary across different age groups. An important criterion for subsequent research is the division of codependent women into groups based on the nature of the relationship with the dependent, the degree of kinship (parents–children, husband–wife, partnerships, close relatives), as well as the social situation that affects the addiction of their loved ones. More in-depth investigations into the nature of relationships between addicts and codependent women, and how social situations mediate addictive behavior, could provide more insight into the phenomenon of codependency. Finally, further exploration of the severity of codependent behavior in different groups of codependent women could help to identify unique features of self-attitude and reflection in these individuals.

## Limitations

It is important to note some limitations of this study. First, the study sample consisted of codependent women aged from 18 to 70 years, which gave us a broad idea of self-attitude and reflection in women at different stages of life, but did not allow us to track any age specifically or dynamics. Additionally, the nature of relationships with an addict was not taken into account. At this stage, we were limited by the sample size, which did not allow more differentiation. Some of the subgroups had fewer than 30 participants, which may have impacted the statistical power of the study.
